# Characteristics of endoscopic and pathological findings of amebic colitis

**DOI:** 10.1186/s12876-021-01941-z

**Published:** 2021-10-09

**Authors:** Bing Yue, Ying Meng, Yanhua Zhou, Haiying Zhao, Yongdong Wu, Ye Zong

**Affiliations:** 1 Department of Pathology, Beijing Friendship Hospital, Capital Medical University, Beijing, China; 2 Department of Gastroenterology, Beijing Friendship Hospital, Capital Medical University, Beijing, 100050 China; 3 National Clinical Research Center for Digestive Diseases, Beijing, China

**Keywords:** Amebic colitis, Endoscopy, Pathology

## Abstract

**Background:**

The clinical features of amoebic colitis resemble those of inflammatory bowel disease (IBD), and therefore the risk of misdiagnosis is very high. The aim of this study was to analyse the characteristics of the endoscopic and pathological findings of amebic colitis and the lessons from our patients, which were useful for diagnosing the amebic colitis timely and avoiding the serious complication.

**Methods:**

We retrospectively reviewed data of all amebic colitis admitted to Beijing Friendship Hospital from January 2015 to January 2020. Cases were diagnosed by clinical presentation, laboratory examinations, and colonoscopy with biopsy and histological examination, no ELISA stool antigen or PCR tests were used.

**Results:**

16 patients were diagnosed with amebic colitis by the colonoscopy accompanied by biopsy and microscopic examination. At first time, 12 (75%) patients were misdiagnosed as IBD. Cecum was the most common site of amebic colitis (100%), and the caecum and rectum were also involved in many lesions (68.75%). Multiple lesions of erosion and/or ulcer were recognized in all patients (100%).The endoscopic findings included multiple irregular shaped ulcers and erosions with surrounding erythema, and the ulcers and erosions were covered by the white or yellow exudates. The intervening mucosae between the ulcers or erosions were normal. The features of rectums can be divided to 2 types: in 6 patients (54.5%), the irregular ulcer or erosions covered with white or yellow exudates were observed in rectum and cecum, and the bloody exudates in rectum were more severe than those in cecum; in other 5 patients (45.5%), rectal lesions were much less severe than those in cecum, the small superficial erosion or reddened mucosa were observed in the rectal ampulla. All patients were diagnosed as detection of amebic trophozoites from HE-stained biopsy specimens. The number of trophozoites ranged from 1/HPF to > 50/HPF. Among 16 cases, mild architectural alteration of colon crypt were observed in 10 cases (62.5%), and serious architectural alteration of colon crypt was found which had crypt branch in 1 case (16.7%). Cryptitis was observed in 12 cases (75%) and its severity was mild or moderate. No crypts abscess was observed in all cases.

**Conclusions:**

The colonoscopy with histological examination are very important to diagnose the amebic colitis. Detect the amoebic trophozoites in the exudates by histological examination is the vital. Sometimes a negative biopsy does not rule out amebiasis, repeated biopsies may be needed to make the diagnosis.

## Background

Amebic colitis, caused by intestinal infection with the parasite, *Entamoeba histolytica*, is a common cause of diarrhea worldwide. The vast majority of amebic infections are asymptomatic, with approximately 10% of those infected progressing to have symptoms [1]. Amebic colitis is the most common symptomatic manifestation, with variable presentation, including watery diarrhea, dysentery, abdominal pain, tenderness and rarely the formation of a tumor like granulation mass referred to as an ameboma [2]. Although the complications are unusual, some complications are very serious such as fulminant necrotizing colitis, toxic megacolon, and fistulizing perianal ulcerations when diagnosis and treatment are not timely. Prevalence is disproportionately higher in developing countries because of poor socioeconomic and sanitation conditions. Areas with the highest rates of amoebic infection include India, Africa, Mexico, and some parts of Central and South America. In developed countries, therefore, amoebiasis is not common. Inflammatory bowel disease (IBD) is common in developed countries and an increasing incidence and prevalence of inflammatory bowel disease has been witnessed in developing countries. Because the clinical features of amoebic colitis resemble those of IBD, and therefore the risk of misdiagnosis is high [3–6]. The consequences of not recognizing amebic colitis can be catastrophic [7, 8]. It may result in administration of steroids or in major intestinal resections for a suspected inflammatory bowel disease, a disease that is relatively simple to treat with metronidazole. Early and accurate diagnosis is extremely important. Some endoscopic and histologic features could be useful for differential diagnosis [9–11]. But in non-endemic countries and area, the amebic colitis is still often misdiagnosed. Thus, the aim of this study was to analyse the characteristics of the endoscopic and pathological findings of amebic colitis and the lessons from our patients, which were useful for diagnosing the amebic colitis and avoiding the serious complications.

## Methods

### Patients

This retrospective study was approved by the Ethic Committee of Beijing Friendship Hospital. 16 adult patients with amebic colitis referred to Beijing Friendship Hospital were included in the study from January 2015 to January 2020.

### Colonoscopy

Colonoscopy was performed for all included patients and the biopsies were done during the colonoscopic procedure. Amebic colitis was defined as detection of amebic trophozoites from HE-stained biopsy specimens.

### Data collection

Clinical features, endoscopic data, pathological findings and effectiveness of treatment were all analyzed.

## Results

### Clinical features of 16 patients with amebic colitis

From January 2015 to January 2020, 16 patients were diagnosed with amebic colitis by the colonoscopy accompanied by biopsy and microscopic examination. Amebic colitis was defined as the presence of amebic trophozoites in biopsy specimens. Among the 16 patients, 15 were males and 1 was female. The age range of the patients was from 31 to 67 years (median 42 years). Symptoms among 16 patients were bloody stools (56.3%, 9/16), abdominal pain or discomfort (62.5%, 10/16), and diarrhea (25.0%, 4/16). And 1 patient had no symptoms, he underwent colonoscopy because of positive fecal occult blood test in routine physical examination. There was no patient with HIV infection, but 1 patient with siphilis. The time from onset to diagnosis was 1 months to 3 years. At first time, 12 (75%, 12/16) patients were misdiagnosed as IBD (8 ulcerative colitis, 2 Crohn disease, and 2 unclassified IBD). There was no complication such as fulminant necrotizing colitis, toxic megacolon, fistulizing perianal ulceration and amebic liver abscess in all patients. Among 10 patients with blood routine examination, the level of hemoglobin decreased in 2 patients (67 g/l and 108 g/l), who had bloody stools with 3–6 times per day for 1–2 year, and others were normal. Unfortunately, the results of stool parasite were negative in all patients.

### Endoscopic findings

**Fig. 1:**
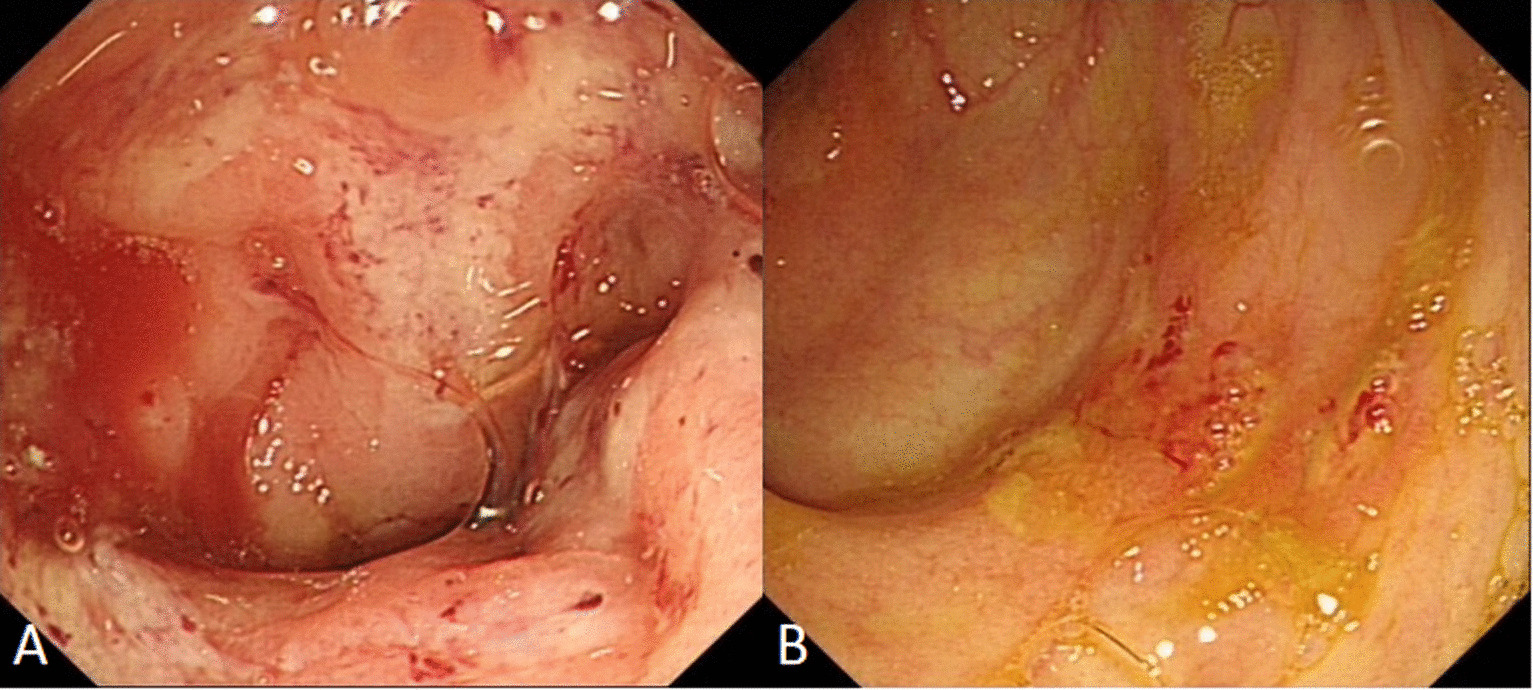
The irregular erosions covered with white exudates were observed in rectum (**a**) and cecum (**b**), and the bloody exudates in rectum were more severe than those in cecum

**Fig. 2:**
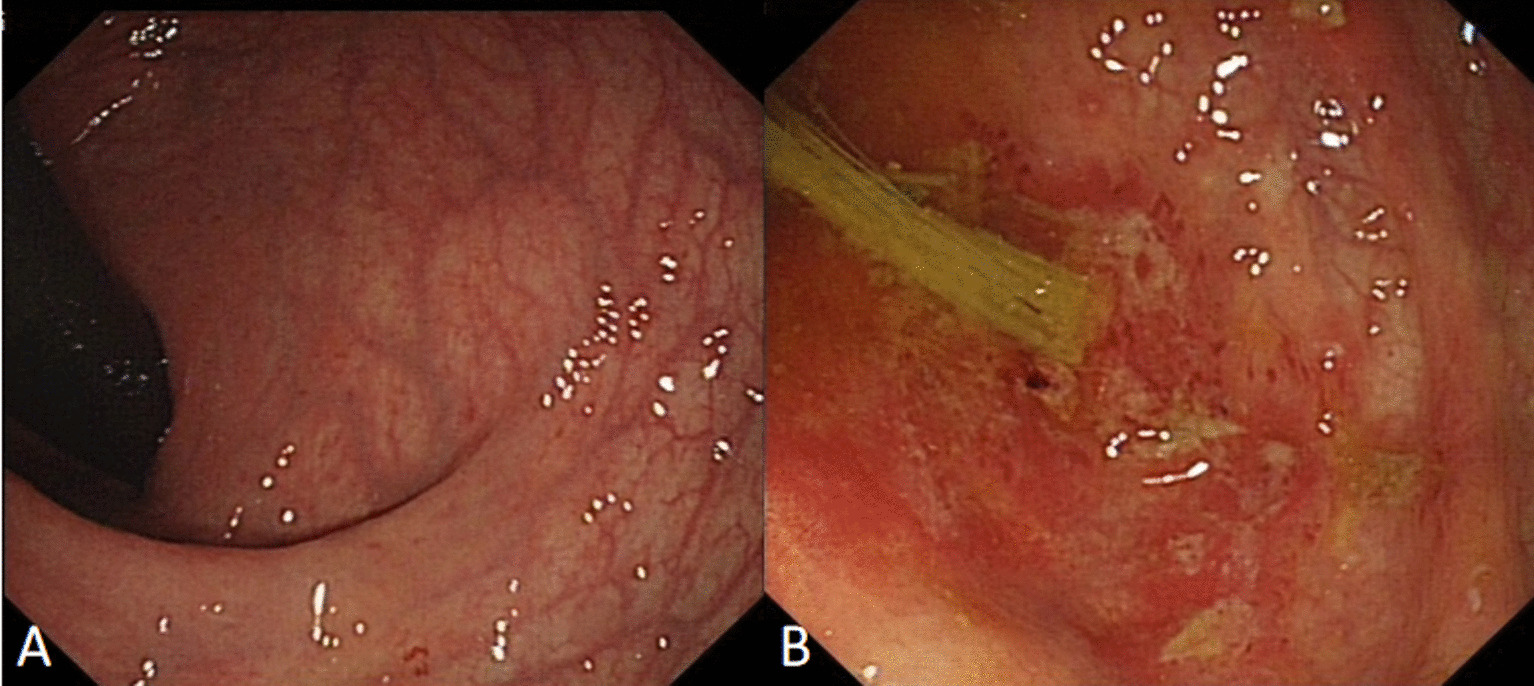
The rectal lesions (**a**) were much less severe than those in cecum (**b**), the small superficial erosion or reddened mucosa were observed in the rectal ampulla (**a**), and the irregular erosions covered with white or yellow exudates were observed in the cecum (**b**)

All patients underwent colonoscopy before diagnosis. 12 patients underwent colonoscopy for more than once before diagnosis: 3 patients for 3 times and 9 patients for twice. The lesion distribution of colitis was as follows: 9 patients (56.2%, 9/16) in cecum and rectum, 2 patients (12.5%, 2/16) in cecum and sigmoid colon, 2 patients (12.5%, 2/16) in cecum and rectosigmoid colon, 2 patients (12.5%, 2/16) in cecum, ascending colon and transverse colon, and 1 (6.3%, 1/16) patient in cecum. Multiple lesions of erosion and/or ulcer were recognized in all patients (100%). The endoscopic findings included multiple irregular shaped ulcers and erosions with surrounding erythema, and the ulcers and erosions were covered by the white or yellow exudates. The intervening mucosae between the ulcers or erosions were normal. 11patients had the lesions involving the rectum, the features of rectums can be divided to 2 types: in 6 patients (54.5%, 6/11), the irregular ulcer or erosions covered with white or yellow exudates were observed in rectum and cecum, and the bloody exudates in rectum were more severe than those in cecum (Fig. 1); in other 5 patients (45.5%, 5/11), rectal lesions were much less severe than those in cecum, the small superficial erosion or reddened mucosa were observed in the rectal ampulla, however, the irregular ulcer or erosions covered with white or yellow exudates were observed in the cecum (Fig. 2). In other 4 patients with multisegmental lesions, the lesions such as the size of ulcer, exudates and erythema around the ulcers, in cecum were more serious than those in other segment. The lesions located only in cecum showed the irregular superficial ulcers covered with the white exudates, and this patient had no apparent symptoms (Table 1).

**Table 1:** Clinical features of 16 patients with amebic colitis

No	Sex	Symptoms	Endoscopic findings
1	M	Blood stools, abdominal pain	Multiple erosion and ulcers in the cecum and the rectum
2	M	Blood stools, abdominal pain	Multiple irregularly shaped ulcers ulcers in the cecum and the rectum
3	M	Diarrhea	The small superficial erosion or reddened mucosa in the rectum, and irregular ulcers in the cecum
4	M	Blood stools, abdominal pain	Multiple irregularly shaped ulcers in the cecum and rectosigmoid colon
5	M	Blood stools, abdominal pain	Multiple irregularly shaped ulcers in the cecum and the rectum
6	M	Abdominal pain, blood stools	Multiple irregularly shaped ulcers in the cecum and the rectum
7	M	No symptoms	Multiple small irregularly shaped ulcers in the cecum
8	M	Blood stools	Multiple irregularly shaped ulcers in the cecum and the sigmoid colon
9	M	Diarrhea	The small superficial erosion or reddened mucosa in the rectum, and irregular ulcers in the cecum
10	M	Blood stools, abdominal pain	Multiple irregularly shaped ulcers in the cecum, ascending colon and transverse colon
11	M	Abdominal discomfort	The small superficial erosion or reddened mucosa in the rectum, and irregular ulcers in the cecum
12	M	Lower abdominal pain	The small superficial erosion or reddened mucosa in the rectum, and irregular ulcers in the cecum
13	M	Diarrhea with mucus or blood	Multiple irregularly shaped ulcers in the cecum and the sigmoid colon
14	M	Abdominal pain	The small superficial erosion and ulcers in the cecum, ascending colon and transverse colon
15	M	Abdominal discomfort	The small superficial erosion or reddened mucosa in the rectum, and irregular ulcers in the cecum
16	F	Blood stools	Multiple irregularly shaped ulcers in the cecum and rectosigmoid colon

### Pathological findings

All patients were diagnosed as detection of amebic trophozoites from HE-stained biopsy specimens. However, there were 12 patients (75%, 12/16) were not diagnosed timely, so these patients underwent colonoscopy for more than once. In this study, all the biopsy specimens were examined again. Actually the amebic trophozoites were observed in necrotic material admixed with mucin, proteinaceous exudate covering the ulcerated mucosa in all patients on every colonoscopic biopsies examination.

**Fig. 3:**
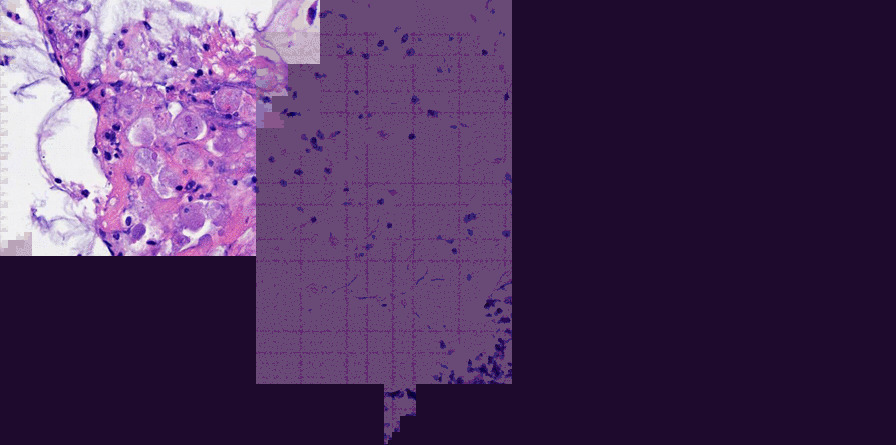
Typical Pathological Findings of amebic colitis: superficial ulcer was located in the lamina propria of mucosa, inflammatory exudates were on the surface of mucosa, and the exudates consisted of fibrin, necrotic material and inflammatory cells. Amoebic trophozoites were observed in inflammatory exudates

**Fig. 4:**
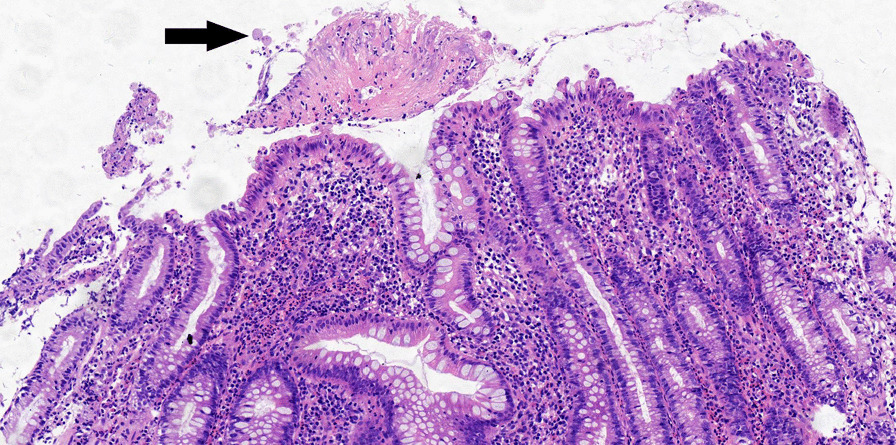
Atypical pathological findings of amebic colitis which was misdiagnosed at first: acute moderate cryptitis with architectural alteration of colon crypt, few inflammatory exudates, and only 3 amoebic trophozoites in the exudates

Among the 16 cases, superficial ulcers which all located in the lamina propria of mucosa were observed by microscopy examination in 8 cases (50%, 8/16). In all cases, various inflammatory exudates were observed on the surface of mucosa, and the exudates consisted of fibrin, necrotic material and inflammatory cells. Amoebic trophozoites were observed mainly in inflammatory exudates or on surface of mucosa. The number of trophozoites ranged from 1 /HPF to > 50/HPF. Tissue invasion was no observed in all cases. Among 16 cases, mild architectural alteration of colon crypt were observed in 10 cases (62.5%, 10/16), and serious architectural alteration of colon crypt was found which had crypt branch in 1 case (16.7%, 1/16). Cryptitis was observed in 12 cases (75%, 12/16) and mainly located in the superficial layer of mucosa, which severity was mild or moderate. No crypts abscess was observed in all cases. There were lymphocytes, neutrophils and eosinophils in the lamina propria of the mucosa. The number of eosinophils in the lamina propria were 5–80/HPF, and the number of eosinophils were more than 50 /HPF in 10cases (62.5%, 10/16) (Figs. 3 and 4).

### Treatment

All patients initially diagnosed with IBD were treated with mesalazine, but this was not effective for any of the patients. There was no administration of steroids for all patients. When the patients was diagnosed with amebic colitis, they were treated with metronidazole 400 mg, three times per day for 14 days. Among 16 patients, 10 patients were followed up. The symptoms were disappeared after the treatment of metronidazole. 2 patients underwent the colonoscopy 1 month after treatment of metronidazole, the results showed the ulcers and erosions were disappeared and the mucosa of colon was normal. 8 patients underwent the colonoscopy 6–12 months after treatment of metronidazole, the results also showed normal appearances.

## Discussions

At present, amebic colitis is not very common in many area and counties. A total of 4366 amoebic dysentery cases were reported without death in China during 2015–2018 and the reported average annual incidence was 0.08/100,000, and the patients were mainly children aged under 5 years (42.28%) [12]. A systematic review of patients with amoebic colitis who received steroids for initially misdiagnosed colitis noted that rapid progression of disease following steroid therapy was common, nearly half of all cases underwent surgical intervention, and 25% of cases died, despite all patients eventually receiving treatment with metronidazole [13]. So amebic colitis is often misdiagnosed in adult at first, and the diagnosis of amebic colitis timely is very important.

Various diagnostic tools exist for the diagnosis of *Entamoeba histolytica* including microscopy, serology, antigen detection, molecular techniques, and colonoscopy with histological examination [14]. Because amebic colitis is not common, the kits for serology assay or antigen detection are not available in many hospital. Examination of stool is a simple and inexpensive investigation in a patient with diarrhea and may reveal trophozoites of *E. histolytica* [15]. However, the results of stool parasite were negative in all patients in our study which is similar to the previous studies [16]. Microscopy requires specialized expertise and is subject to operator error, and the stool should be checked as soon as possible, so the diagnostic sensitivity and specificity of microscopic examination to detect *E. histolytica* in stool is considered low. Actually, most of time, amebic colitis is one of the differential diagnosises of colonic ulcers. In many non-endemic countries and areas, the colonoscopy with histological examination is available and effective [17, 18] for diagnosing the amebic colitis. Some studies suggest that on endoscopy it is difficult to distinguish amebic colitis from IBD, CMV colitis, intestinal tuberculosis, and pseudomembranous colitis due to C. difficile infection [19, 20].Other studies reported the endoscopic procedure may contribute to early diagnosis of the disease and to the prevention of serious complications. Nagata et al. investigated the sensitivity and specificity of endoscopic findings that were significantly associated with amebic colitis were: cecal lesions, multiple number of lesions, presence of aphthae or erosions and presence of exudate. Multivariate analysis revealed that the best combination of findings to predict amebic colitis was the presence of cecal lesions, multiple lesions, and exudates [9]. Lee et al. observed distinct differences in findings based on right-side amebic colitis versus proctosigmoiditis, the colonoscopic findings of right-sided colitis included aphthae or erosions, ulcers, exudates, or edematous swollen mucosa in cecum, and findings for proctosigmoiditis were edematous swollen mucosa with bloody exudate [21]. In our study, cecum was the most common site of amebic colitis, and the caecum and rectum were also involved in many lesions (11/16, 68.75%). The features of the lesions in rectums had 2 types, in some patients, the irregular ulcer or erosions covered with white or yellow exudates were observed in rectum and cecum, and the bloody exudates in rectum were more severe than those in cecum; in other patients, rectal lesions were much less severe than those in cecum, the small superficial erosion or reddened mucosa were observed in the rectal ampulla, however, the irregular ulcer or erosion covered with white or yellow exudates were observed in the cecum, which was different from the report of Lee [21]. The features of the typical lesions in our study were similar to the previous reports [10, 22], which included multiple irregular shaped ulcers and erosions with surrounding erythema, and the ulcers and erosions were covered by the white or yellow exudates which were named “dirty ulcer”.

The diagnostic value of the colonoscopy lies in the ability to take biopsies and microscopically identify intestinal amoebiasis. The pathological findings is vital in diagnosing amebic colitis [22]. In our studies, the amebic trophozoites were observed in the HE-stained biopsy specimens in all patients, although the biopsy specimens were misdiagnosed at first in 12 patients. The reasons of the misdiagnosis are: (1) In some specimens, the number of the trophozoites was less and the pathologist observed not carefully, which led to misdiagnose. (2) Some pathologists had no experience in diagnosing amoebic enteritis, they often spend time observing the structure of the colonic mucosa and changes of epithelium, while ignored the exudates, which were the most common place of trophozoites. In one case of our study, the active inflammation in colonic mucosa and the disordered structure of crypts were significant, which was similar with IBD, and the trophozoites were very less in the exudates, so the pathologist made a misdiagnosis at first. In our study, in addition to the observation of amoebic trophozoites, there were some characteristics of pathologic findings in amebic colitis: (1) The ulcer was generally superficial. (2) The surface of the ulcer was often covered with the inflammatory exudates. (3) The degree of the structure disorder was relatively mild, and generally there was no branch. (In our study, there was only one case which had crypt branch). (4) Cryptitis was mild, and generally located in the superficial layer of mucosa. (5) Crypt abscess was no common. Prathap and Gilman reported the crypt abscesses were not found in any of the 53 rectal biopsies in acute amebic colitis [23]. (6) There were a lot of eosinophil in the mucosa lamina propria, but it was not specific. All of the histologic features could be useful in differentiating amebiasis from IBD and other colitis.

This study had some limitations. First, Beijing is no-epidemic area of amebiasis, so the number of patients with amebic colitis in our study was small. Second, the selection bias was present because there were no patients with fulminant amebic colitis or amebic abscess in our study, which meant there were no acute serious cases in our study.

## Conclusion

Our study showed the colonoscopy with histological examination are very important to diagnose the amebic colitis. When the characteristic of the colonoscopy is similar to that of the amebic colitis, the biopsy with careful pathological examination is necessary. Detect the amoebic trophozoites in the exudates is the vital.

## Data Availability

Some data analysed during this study are included in this manuscript (Table 1). And other datasets used and/or analysed during the current study available from the corresponding author on reasonable request.

## Abbreviation

IBD: Inflammatory bowel disease
